# Radiation Therapy after Radical Prostatectomy for Prostate Cancer: Evaluation of Complications and Influence of Radiation Timing on Outcomes in a Large, Population-Based Cohort

**DOI:** 10.1371/journal.pone.0118430

**Published:** 2015-02-23

**Authors:** Sarah E. Hegarty, Terry Hyslop, Adam P. Dicker, Timothy N. Showalter

**Affiliations:** 1 Division of Biostatistics, Kimmel Cancer Center & Jefferson Medical College, Thomas Jefferson University, Philadelphia, Pennsylvania, United States of America; 2 Department of Biostatistics & Bioinformatics, Duke University School of Medicine, Durham, North Carolina, United States of America; 3 Department of Radiation Oncology, Jefferson Medical College and Kimmel Cancer Center, Thomas Jefferson University, Philadelphia, Pennsylvania, United States of America; 4 Department of Radiation Oncology, University of Virginia School of Medicine, Charlottesville, Virginia, United States of America; Innsbruck Medical University, AUSTRIA

## Abstract

**Purpose:**

To evaluate the influence of timing of salvage and adjuvant radiation therapy on outcomes after prostatectomy for prostate cancer.

**Methods:**

Using the Surveillance, Epidemiology, and End Results-Medicare linked database, we identified prostate cancer patients diagnosed during 1995–2007 who had one or more adverse pathological features after prostatectomy. The final cohort of 6,137 eligible patients included men who received prostatectomy alone (n = 4,509) or with adjuvant (n = 894) or salvage (n = 734) radiation therapy. Primary outcomes were genitourinary, gastrointestinal, and erectile dysfunction events and survival after treatment(s).

**Results:**

Radiation therapy after prostatectomy was associated with higher rates of gastrointestinal and genitourinary events, but not erectile dysfunction. In adjusted models, earlier treatment with adjuvant radiation therapy was not associated with increased rates of genitourinary or erectile dysfunction events compared to delayed salvage radiation therapy. Early adjuvant radiation therapy was associated with lower rates of gastrointestinal events that salvage radiation therapy, with hazard ratios of 0.80 (95% CI, 0.67–0.95) for procedure-defined and 0.70 (95% CI, 0.59, 0.83) for diagnosis-defined events. There was no significant difference between ART and non-ART groups (SRT or RP alone) for overall survival (HR = 1.13 95% CI = (0.96, 1.34) p = 0.148).

**Conclusions:**

Radiation therapy after prostatectomy is associated with increased rates of gastrointestinal and genitourinary events. However, earlier radiation therapy is not associated with higher rates of gastrointestinal, genitourinary or sexual events. These findings oppose the conventional belief that delaying radiation therapy reduces the risk of radiation-related complications.

## Introduction

There are over 230,000 new prostate cancer (PC) diagnoses in the United States each year [1], and one-third of affected men choose to undergo radical prostatectomy (RP) [2]. One in 5 PC patients recur after RP [3], and recurrence rates are higher, 40–60%, for patients with one or more adverse pathological features (APFs), including: extracapsular extension (ECE), seminal vesicle invasion (SVI) or positive surgical margin (PSM) [4]. For PC patients who are at higher risk of recurrence, adjuvant radiation therapy (ART) to the prostate bed may be offered based on adverse pathologic factors (APFs) alone without a detectable prostate specific antigen (PSA) blood level after surgery. ART has been shown in randomized trials to improve PSA-relapse free survival [5–7], distant metastasis-free survival and overall survival [8], compared to observation.

However, less than 20% of qualifying patients in the United States receive ART [9–11]. Many clinicians prefer to follow patients after RP and to deliver salvage radiation therapy (SRT) to the prostate bed when the PSA level rises [12], a potentially curative strategy [13]. The primary reasons cited for choosing selective SRT over ART include the perceived toxicity of post-RP RT, the potential overtreatment of patients with ART who may not have recurred after RP, the importance of delaying RT to allow time for recovery of urinary and sexual function after RP, and the presumed equivalence of ART and SRT strategies for cancer control [12]. A national survey of urologists and radiation oncologists demonstrated that providers’ perceptions of the risk of radiation-induced urinary complications significantly impacted the likelihood of recommending ART after RP for patients with APFs [14].

Although the risk of toxicity influences ART decisions, the available evidence on this topic is limited. In a randomized controlled trial of ART versus observation conducted by the Southwest Oncology Group (SWOG), which included conventional RT techniques, complete urinary incontinence was more common after ART than after RP alone (6.5% versus 2.8%, p = 0.11) [5]. However, two other randomized trials did not show an increase in urinary incontinence after ART compared to RP alone [6,15].

Given the relevance of toxicity risk, and the influence of radiation timing, to decisions regarding post-RP RT, it is important to provide additional evidence. Trials designed to directly compare ART to selective use of SRT are ongoing, but results are not expected for a decade or longer. This analysis of a population-based cohort was designed to evaluate the comparative effectiveness of early post-prostatectomy RT (ART), compared to prostatectomy alone and delayed RT (SRT), for prostate cancer patients who qualify for adjuvant RT based on APFs. Important secondary objectives include evaluation of predictors of RT utilization after prostatectomy, the influence of RT timing on risk of complication events, and the impact of delaying RT on survival among high-risk patients.

## Materials and Methods

### Data Source

The Surveillance, Epidemiology, and End Results (SEER) database contains data from population-based tumor registries in several regions of the United States and represents approximately 26% of the total United States population [16]. The SEER-Medicare linked database matches administrative claims data from Medicare with subjects from the SEER registry for United States citizens ages 65 years and older who are Medicare beneficiaries [17]. The SEER-Medicare database has been used previously to compare outcomes after intensity-modulated radiation therapy versus conformal radiation therapy in the post-prostatectomy setting [18,19]. The research was conducted according to the data use agreement of SEER-Medicare, and the manuscript was approved by SEER-Medicare prior to submission. Patient records were anonymized and de-identified prior to analysis.

### Study Cohort

**Fig 1:**
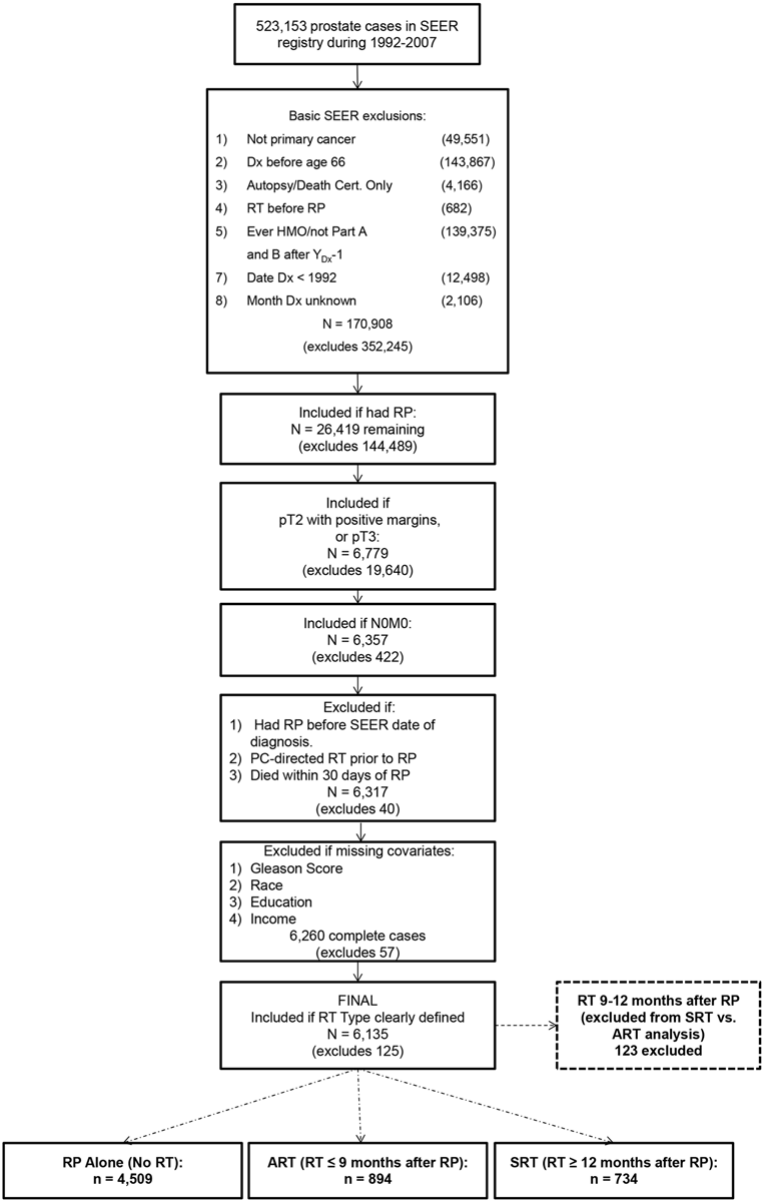
Flow diagram designed to illustrate the development of the study cohort through stepwise exclusions using SEER registry data elements and administrative codes from the Medicare database.

After Thomas Jefferson University institutional review board approval, the SEER-Medicare database was searched to identify 523,153 men who were diagnosed with prostate cancer between 1992 and 2007, who were enrolled continuously in both Parts A and B of Medicare, and who had at least 30 days of observation after RP. The cohort was reduced to 170,908 prostate cancer patients after excluding individuals with a previous cancer diagnosis, those who were diagnosed before age 66 years, men who were enrolled in a health maintenance organization (HMO) at any time starting 1 year prior to their prostate cancer diagnosis, and other reasons that would limit data availability (Fig. 1). Of these men, a total of 26,419 received RP. From this group, a cohort of 6,357 men were considered eligible for ART based on the presence of one or more APFs in the RP surgical specimen (pT3 or pT2 with positive margins) and no evidence of regional (N0) or distant (M0) metastases. A further 29 men were excluded due to prior PC-directed RT or due to a record for RP prior to diagnosis. Eleven were excluded due to death within 30 days of RP, as 30-days post-RP was the starting point for the complication events analyses. Subsequent exclusions were made due to missingness in potential confounders: tumor grade, race, education and income information. Treatment, complication and comorbidity information were extracted from the Medicare administrative claims, using Current Procedural Terminology (CPT), Healthcare Common Procedure Coding System (HCPCS) and associated International Classification of Diseases (ICD) codes (see S1 Table). The administrative claims codes used in the current analysis were adapted from several prior published reports [20–23]. Among these men, a total of 4,509 received RP alone, and 1,751 received RP followed by postoperative RT (Fig. 1).

In order to evaluate the hypothesis that delaying the delivery of post-RP RT influences outcomes, the group of patients who received RT was further classified based upon length of time from RP to RT as ART (RT within 9 months of RP, n = 894) and delayed SRT (RT 12+ months after RP, n = 734). The SEER-Medicare database does not provide data regarding the intent of RT, nor technical details regarding the RT fields, so RT timing was used as a surrogate classifier of adjuvant versus salvage therapy. The investigators chose this ART/SRT classification system empirically based upon impressions regarding clinicians’ practices regarding ART timing, with support from the results of a prior national survey [14]. Patients receiving RT between 9 and 12 months after RP (n = 123) were excluded from analyses of ART and SRT to limit misclassification of ART versus SRT.

### Outcomes

First complication events were evaluated after RP for each of four categories: erectile dysfunction (ED), gastrointestinal (GI), urinary incontinence (UI), and urinary non-incontinence (UN). Complication events were identified from Medicare claims based upon HCPCS/CPT-4 procedure codes and ICD-9 diagnosis codes (S1 Table); procedure code-defined and diagnosis code-defined events were analyzed separately. The observation period for events began 30 days after RP in order to ignore acute complications. Event rates for RP followed by RT include complications attributable to either or both interventions, while the RP alone cohort serves as a control for comparisons. Times to complication event were measured from 30 days post-RP to the first event date or censored at death or end of study, December 31, 2008. Overall and prostate cancer-specific survival times were measured from date of diagnosis to death or censoring at the end of study. Further details about control variables are provided in (S1 Text).

### Statistical Analysis

Patient characteristics among RP alone, ART and SRT groups were compared using chi-square test. Multivariable analysis (MVA) with polytomous logistic regression was performed to evaluate predictors of ART utilization (versus either no RT or SRT). This model included the following covariates: race, Hispanic origin, marital status, census-tract % high school completion, census-tract median income, population density, SEER region, year of diagnosis, pT stage, tumor grade, margin status, age at diagnosis, comorbidity score, androgen deprivation therapy receipt, surgery type and indicators for having a history of ED, GI, UI and UN events (based on procedure and diagnosis codes combined) in the year prior to RP. From this model, a propensity score weight was calculated as the inverse predicted probability of being in one’s treatment group; this weight was then adjusted by the relative sample size of each treatment group.[24] Propensity score weighting was used to adjust for potential differences in baseline characteristics between patients in the RP alone and RP plus RT groups, and characteristics were compared again using the chi-square test.

First complication events (based on either procedure or diagnosis code) were reported in events/100 person years within each treatment group as fixed at the end of study. Adjustment for potential confounders was performed by propensity score weighting [25]. 95% confidence intervals of adjusted rate ratios were calculated via propensity-weighted Poisson regression incorporating an offset of complication-free survival time [26]. The investigators designed the Poisson regression model in order to estimate and compare event rates among the three cohorts. All comparison tests were 2-tailed, and the threshold for statistical significance was p = 0.05.

The primary analysis was comprised of multivariate Cox proportional hazards models of time to first event were performed for each class of events (ED, GI, UI, UN) with procedure code-defined and diagnosis code-defined classes being considered separately. Radiotherapy type—RP alone, ART or SRT—was included as a time-dependent variable [27]. The inclusion of RT as a time-dependent variable in these models allowed for an individual’s RT status to change over time. That is, all men began the study period in the RP alone group and then, over the course of the follow-up period, some men switch to the RP and ART group, others to the RP and SRT group, while others remain in the RP alone group for the duration. Considering the addition of ART and SRT separately allowed for the evaluation of the influence of RT timing on the probability of complication events. All models included the potential confounders contained in Table 1 and were weighted by the propensity score. Hazard ratios were calculated with 95% confidence intervals and p values were considered significant if < 0.05.

**Table 1:** Demographic and clinical characteristics of subjects who received radical prostatectomy (RP) alone, RP followed by adjuvant radiation therapy, or RP followed by salvage radiation therapy.

Predictor	Total		Radiotherapy Use						p
			RP only		ART (<9mo)		SRT (12mo+)		
	N	Col %	N	Col %	N	Col %	N	Col %	
**Total**	6,137	100	4,509	100	894	100	734	100	
**Pathological T-Stage**									<0.001
T2	1,856	30.2	1,494	33.1	181	20.2	181	24.7	
T3a	3,140	51.2	2,294	50.9	467	52.2	379	51.6	
T3b	1,141	18.6	721	16.0	246	27.5	174	23.7	
**Margin Status**									0.259
Uninvolved	3,175	51.7	2,308	51.2	468	52.3	399	54.4	
Involved	2,962	48.3	2,201	48.8	426	47.7	335	45.6	
**Gleason Score**									<0.001
≤7	4,233	69.0	3,384	75.0	431	48.2	418	56.9	
8+	1,904	31.0	1,125	25.0	463	51.8	316	43.1	
**Age at Diagnosis**									0.002
66–69	3,234	52.7	2,306	51.1	507	56.7	421	57.4	
70–74	2,357	38.4	1,784	39.6	313	35.0	260	35.4	
75–79	546	8.9	419	9.3	74	8.3	53	7.2	
**Comorbidity Score**									0.027
0	3,765	61.3	2,719	60.3	565	63.2	481	65.5	
1	1,637	26.7	1,222	27.1	231	25.8	184	25.1	
2+	735	12.0	568	12.6	98	11.0	69	9.4	
**Diagnosis Year**									<0.001
1995–1999	1,576	25.7	1,103	24.5	228	25.5	245	33.4	
2000–2004	2,696	43.9	1,940	43.0	382	42.7	374	51.0	
2005–2007	1,865	30.4	1,466	32.5	284	31.8	115	15.7	
**Race**									0.126
White	5,509	89.8	4,051	89.8	793	88.7	665	90.6	
Black	368	6.0	281	6.2	51	5.7	36	4.9	
Other/Unspecified	260	4.2	177	3.9	50	5.6	33	4.5	
**Hispanic Ethnicity**									0.399
Non-Hispanic	5,759	93.8	4,220	93.6	845	94.5	694	94.6	
Hispanic	378	6.2	289	6.4	49	5.5	40	5.4	
**Marital Status**									0.381
Not Married	868	14.1	658	14.6	121	13.5	89	12.1	
Married	5,113	83.3	3,733	82.8	751	84.0	629	85.7	
Unknown	156	2.5	118	2.6	22	2.5	16	2.2	
**HS Education Attainment**									0.164
<75%	1,068	17.4	804	17.8	156	17.4	108	14.7	
75–84.99%	1,387	22.6	1,037	23.0	196	21.9	154	21.0	
85–89.99%	1,196	19.5	878	19.5	164	18.3	154	21.0	
90%+	2,486	40.5	1,790	39.7	378	42.3	318	43.3	
**Median Household Income**									0.446
<35K	1,233	20.1	928	20.6	169	18.9	136	18.5	
35K-44K	1,305	21.3	966	21.4	175	19.6	164	22.3	
45K-59K	1,526	24.9	1,105	24.5	240	26.8	181	24.7	
60K+	2,073	33.8	1,510	33.5	310	34.7	253	34.5	
**Population Density**									0.802
Urban	6,030	98.3	4,432	98.3	879	98.3	719	98.0	
Rural	107	1.7	77	1.7	15	1.7	15	2.0	
**Treatment Region**									0.140
West	3,771	61.4	2,766	61.3	557	62.3	448	61.0	
Midwest	1,100	17.9	825	18.3	134	15.0	141	19.2	
Northeast	573	9.3	406	9.0	100	11.2	67	9.1	
South	693	11.3	512	11.4	103	11.5	78	10.6	
**Radical Prostatectomy Type**									<0.001
Open	5,250	85.5	3,799	84.3	769	86.0	682	92.9	
MIRP	887	14.5	710	15.7	125	14.0	52	7.1	
**Androgen Deprivation Therapy**									<0.001
No	4,371	71.2	3,654	81.0	396	44.3	321	43.7	
Yes	1,766	28.8	855	19.0	498	55.7	413	56.3	
**History of ED**									0.180
No	5,475	89.2	4,006	88.8	813	90.9	656	89.4	
Yes	662	10.8	503	11.2	81	9.1	78	10.6	
**History of GI**									0.031
No	4,836	78.8	3,517	78.0	719	80.4	600	81.7	
Yes	1,301	21.2	992	22.0	175	19.6	134	18.3	
**History of UI**									0.102
No	5,279	86.0	3,860	85.6	769	86.0	650	88.6	
Yes	858	14.0	649	14.4	125	14.0	84	11.4	
**History of UN**									0.978
No	5,122	83.5	3,765	83.5	744	83.2	613	83.5	
Yes	1,015	16.5	744	16.5	150	16.8	121	16.5	

ART = adjuvant radiation therapy; SRT = adjuvant radiation therapy; HS = high school; MIRP = minimally-invasive radical prostatectomy; ED = erectile dysfunction; GI = gastrointestinal; UI = urinary incontinence; UN = urinary non-incontinence

The impact of radiation on overall and prostate cancer specific survival was evaluated using multivariable Cox proportional hazard models with radiation type (RP only, ART, or SRT) as a time-dependent covariate. The model was weighted by propensity score and adjusted for various clinical, demographic and socioeconomic covariates including: pT stage, tumor grade, surgical margins, age at diagnosis, surgery type, use of androgen deprivation therapy (ADT) at any time, race, ethnicity, median household income, education level, SEER region, year of diagnosis, marital status, population density, comorbidity score and history variables.

## Results

**Fig 2:**
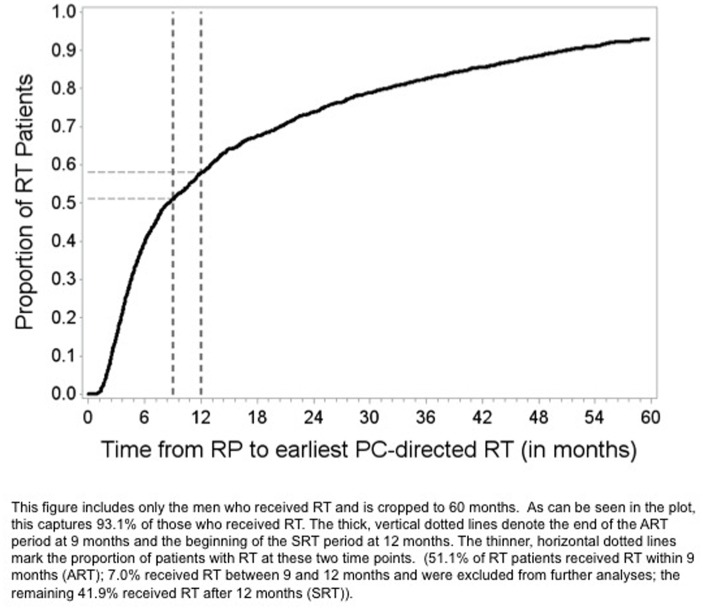
Time from radiation therapy to radical prostatectomy (RP), in months, for the study cohort of men in the SEER-Medicare database who qualified for adjuvant radiation therapy based upon the presence of one of more adverse pathological feature in the prostatectomy specimen.

The cohort was comprised of a total of 6,137 PC subjects who received RP and were eligible for ART. The cohort is further categorized as RP alone (n = 4,509), ART (n = 894) or SRT (n = 734). RT was delivered for a total of 26.5% of subjects at a median of 7.5 months (Fig. 2). ART was delivered for 894 of these subjects (14.6%). Among those who did not receive ART, 14.0% later received SRT. Median follow up from diagnosis was 64 months, 62.9 months, and 84.2 months for the RP alone, RP and ART, and RP and SRT cohorts, respectively. Clinical and demographic characteristics for the RP alone, RP and ART, and RP and SRT cohorts are summarized in Table 1. On univariable analysis, significant differences were found between treatment groups with regards to pT stage, tumor grade, age at diagnosis, comorbidity score, diagnosis year, surgery type, use of ADT at any time, and history of GI. However, after propensity-score weighting only year of diagnosis remained significant (results not shown).

On MVA, predictors of ART utilization included: pT stage, tumor grade, margin status, comorbidity score, age at diagnosis, use of ADT, and year of diagnosis. ART was more likely in patients with more advanced disease with regards to pT stage or APFs than RP alone or SRT. The odds of ART use over RP alone were much higher in those who also received ADT at any time during the study period (RP alone vs. ART: OR = 0.23 (0.20, 0.28) p <0.001) (Table 2).

**Table 2:** Multivariable analysis with polytomous logistic regression performed to evaluate predictors of ART utilization versus no RT or SRT.

	RP alone vs. ART			SRT vs. ART			
	OR	95% CI	p	OR	95% CI	p	p
**Race**							0.641
Black vs. White	1.04	(0.73, 1.48)	0.831	0.89	(0.55, 1.43)	0.627	
Other/Unspecified vs. White	0.79	(0.55, 1.14)	0.205	0.94	(0.59, 1.51)	0.809	
**Hispanic Ethnicity**							0.314
Hispanic vs. Non-Hispanic	1.30	(0.92, 1.86)	0.139	1.15	(0.72, 1.82)	0.561	
**Marital Status**							0.447
Not Married vs. Married	1.14	(0.91, 1.43)	0.266	0.92	(0.68, 1.24)	0.581	
Unknown vs. Married	0.90	(0.55, 1.47)	0.659	0.97	(0.50, 1.88)	0.920	
**HS Education Attainment**							0.117
75–84.99% vs. <75%	1.08	(0.82, 1.43)	0.571	1.13	(0.79, 1.62)	0.513	
85–89.99% vs. <75%	1.07	(0.79, 1.47)	0.655	1.42	(0.95, 2.13)	0.087	
90%+ vs. <75%	0.89	(0.65, 1.23)	0.492	1.33	(0.88, 2.03)	0.178	
**Median Household Income**							0.538
35K-44K vs. <35K	0.96	(0.74, 1.26)	0.790	1.01	(0.71, 1.41)	0.977	
45K-59K vs. <35K	0.87	(0.65, 1.15)	0.322	0.83	(0.58, 1.19)	0.308	
60K+ vs. <35K	1.05	(0.76, 1.45)	0.757	0.90	(0.60, 1.36)	0.616	
**Population Density**							0.985
Rural vs. Urban	0.98	(0.53, 1.82)	0.958	1.04	(0.49, 2.22)	0.923	
**Treatment Region**							0.156
Midwest vs. West	1.31	(1.04, 1.66)	0.023	1.23	(0.92, 1.65)	0.167	
Northeast vs. West	0.81	(0.62, 1.06)	0.120	0.90	(0.63, 1.29)	0.574	
South vs. West	1.00	(0.76, 1.30)	0.968	1.04	(0.74, 1.48)	0.809	
**Year of Diagnosis**							<0.001
2000–2004 vs. 1995–1999	0.95	(0.78, 1.17)	0.633	0.99	(0.77, 1.27)	0.930	
2005–2007 vs. 1995–1999	0.85	(0.67, 1.07)	0.164	0.43	(0.32, 0.60)	<0.001	
**Pathological T-Stage**							<0.001
T3a vs. T2	0.50	(0.40, 0.63)	<0.001	0.64	(0.47, 0.86)	0.003	
T3b vs. T2	0.31	(0.23, 0.43)	<0.001	0.48	(0.32, 0.73)	<0.001	
**Gleason Score**							<0.001
8+ vs. ≤7	0.45	(0.39, 0.53)	<0.001	0.69	(0.56, 0.85)	<0.001	
**Margins Status**							<0.001
Involved vs. Uninvolved	0.45	(0.36, 0.56)	<0.001	0.63	(0.48, 0.84)	0.001	
**Age at Diagnosis**							<0.001
70–74 vs. 65–69	1.36	(1.16, 1.61)	<0.001	0.98	(0.79, 1.21)	0.840	
75–79 vs. 65–69	1.67	(1.24, 2.25)	<0.001	0.97	(0.65, 1.44)	0.865	
80+ vs. 65–69	2.00	(0.77, 5.19)	0.152	0.71	(0.18, 2.91)	0.637	
**Comorbidity Score**							0.027
1 vs. 0	1.14	(0.95, 1.37)	0.149	0.96	(0.76, 1.21)	0.740	
2+ vs. 0	1.25	(0.97, 1.61)	0.079	0.85	(0.60, 1.19)	0.331	
**Androgen Deprivation Therapy**							<0.001
Yes vs. No	0.23	(0.20, 0.28)	<0.001	1.14	(0.93, 1.42)	0.215	
**Radical Prostatectomy Type**							0.064
MIRP vs. Open	1.10	(0.85, 1.41)	0.473	0.74	(0.50, 1.08)	0.116	
**History of ED**							0.130
Yes vs. No	1.24	(0.95, 1.62)	0.113	1.40	(1.00, 1.97)	0.049	
**History of GI**							0.363
Yes vs. No	1.07	(0.88, 1.30)	0.494	0.92	(0.72, 1.19)	0.538	
**History of UI**							0.989
Yes vs. No	1.00	(0.79, 1.28)	0.971	0.98	(0.71, 1.36)	0.920	
**History of UN**							0.761
Yes vs. No	1.08	(0.87, 1.35)	0.488	1.03	(0.77, 1.37)	0.841	

ART = adjuvant radiation therapy; SRT = adjuvant radiation therapy; RP = radical prostatectomy; HS = high school; MIRP = minimally-invasive radical prostatectomy; ED = erectile dysfunction; GI = gastrointestinal; UI = urinary incontinence; UN = urinary non-incontinence

Propensity score-adjusted complication rates were compared for RP alone, RP and ART, and RP and SRT (Table 3). The addition of ART or SRT after RP was not associated with higher rates of ED events, compared to RP alone. Rates of GI events were higher among patients who received ART or SRT than RP alone. ART was associated with higher rates of GU nonincontinence events than RP alone or RT followed by SRT (Table 3).

**Table 3:** Propensity score-adjusted complication rates, by class, for radical prostatectomy alone or in combination with ART (≤ 9 months after RP) or SRT (≥ 12 months after RP).

	No RT n* = 4,521	ART n* = 908	SRT n* = 724	ART vs. No RT	SRT vs. No RT	ART vs. SRT
Complication Class	Adjusted Rate (Events/100 Person Years)	Adjusted Rate (Events/ 100 Person Years)	Adjusted Rate (Events/ 100 Person Years)	Adjusted Rate Ratio (95% CI)	Adjusted Rate Ratio (95% CI)	Adjusted Rate Ratio (95% CI)
**ED**						
Procedures	2.07	1.80	2.02	0.87 (0.69, 1.09)	0.98 (0.77, 1.24)	0.89 (0.66, 1.21)
Diagnoses	12.73	11.01	13.04	0.87 (0.78, 0.96)	1.02 (0.91, 1.15)	0.84 (0.73, 0.98)
**GI**						
Procedures	13.87	16.02	17.06	1.15 (1.05, 1.27)	1.23 (1.11, 1.36)	0.94 (0.83, 1.07)
Diagnoses	9.90	12.68	12.96	1.28 (1.16, 1.42)	1.31 (1.17, 1.46)	0.98 (0.85, 1.12)
**GU-Incontinence**						
Procedures	5.88	6.58	6.74	1.12 (0.98, 1.27)	1.15 (1.00, 1.32)	0.98 (0.82, 1.16)
Diagnoses	9.67	11.30	9.30	1.17 (1.05, 1.30)	0.96 (0.85, 1.09)	1.21 (1.05, 1.41)
**GU- NonIncontinence**						
Procedures	3.78	5.51	3.58	1.46 (1.26, 1.69)	0.95 (0.79, 1.14)	1.54 (1.24, 1.91)
Diagnoses	7.39	9.39	8.46	1.27 (1.13, 1.43)	1.14 (1.00, 1.31)	1.11 (0.95, 1.30)

*Propensity score-weighted sample sizes.

Multivariate Cox proportional hazards models with RT as a time-dependent covariate were performed for GI, ED, GU incontinence and GU non-incontinence events (see S2–S5 Tables), and summary results of comparisons are shown in Table 4. ART and SRT were associated with higher rates of GI and GU, but not ED, events than RP alone. Earlier treatment with ART was associated with lower rates of GI events than SRT, and no increase in GU events (Table 4).

**Table 4:** Summary of findings from multivariable models of complication with RT as time-dependent covariate.

		ART vs. RP alone			SRT vs. RP alone			ART vs. SRT		
Complication Class	n	HR	95% CI	p	HR	95% CI	p	HR	95% CI	p
**ED**										
Procedures	634	0.99	(0.78, 1.24)	0.896	0.54	(0.26, 1.14)	0.104	1.82	(0.85, 3.91)	0.125
Diagnoses	2,770	0.87	(0.77, 0.99)	0.033	0.77	(0.55, 1.07)	0.113	1.14	(0.81, 1.61)	0.460
**GI**										
Procedures	3,146	1.17	(1.06, 1.29)	0.003	1.46	(1.25, 1.69)	<0.001	0.80	(0.67, 0.95)	0.011
Diagnoses	2,582	1.35	(1.21, 1.50)	<0.001	1.93	(1.67, 2.23)	<0.001	0.70	(0.59, 0.83)	<0.001
**GU-Incontinence**										
Procedures	1,701	1.22	(1.05, 1.41)	0.008	1.40	(1.12, 1.76)	0.004	0.87	(0.67, 1.12)	0.281
Diagnoses	2,395	1.29	(1.13, 1.46)	<0.001	1.33	(1.04, 1.69)	0.022	0.97	(0.75, 1.26)	0.818
**GU- Non-Incontinence**										
Procedures	1,148	1.71	(1.45, 2.01)	<0.001	1.42	(1.02, 1.97)	0.039	1.21	(0.85, 1.71)	0.293
Diagnoses	1,967	1.48	(1.29, 1.68)	<0.001	1.46	(1.15, 1.85)	0.002	1.01	(0.78, 1.31)	0.926

Full models for each complication class displayed in Online Supplementary materials.

A total of 981 men died during the observation period; 229 of these deaths were attributed to prostate cancer in the SEER registry. Both overall and prostate cancer-specific survival was worse in the RT groups compared to RP alone. There was no significant difference between ART and non-ART groups (SRT or RP alone) for overall survival (HR = 1.13 95% CI = (0.96, 1.34) p = 0.148). Prostate cancer specific survival was significantly shorter for the ART group compared to the non-ART groups (HR = 1.88 95% CI = (1.35, 2.61) p < 0.001). There was no significant difference in the overall survival of those with ART compared to those with SRT (HR = 0.88 95%CI = (0.68, 1.13) p = 0.305). However, there was a significant difference in the prostate cancer-specific survival of the two RT groups, with a survival benefit seen in the ART group compared to the SRT group (HR = 0.64 95% CI = (0.42, 0.97) p = 0.036).

## Discussion

In this analysis of a large, population-based cohort of patients from the SEER-Medicare database, 14.3% of patients eligible for ART after RP received RT within 9 months after RP and another 11.7% received delayed SRT; a further 2.0% received RT between 9 and 12 months after RP and were excluded from further analyses. Two sets of adjusted analyses, a Poisson regression model and multivariate Cox proportional hazards models with RT as a time-varying covariate, were performed to estimate the occurrence of events after post-RP RT in this cohort and to evaluate the influence of RT timing on outcomes. Observed rates of GI and some GU events, but not ED, were higher in the ART and SRT groups compared to RP alone. Adjusted analyses evaluating RT as a time-dependent covariate revealed that early treatment with ART was associated with lower rates of GI events, and no difference in GU or ED events. There was no overall survival difference observed between ART and non-ART groups.

The current study showed an increased risk of GI and GU, but not ED, events with the addition of ART or SRT after RP. The increase in GI and GU events is consistent with the published literature [12,28,29], and the potential benefits of ART and SRT must be balanced against the incremental risk of GI and GU side effects. The delivery of RT after RP was not associated with increased rate of procedures for ED-related events, which is consistent with the available evidence. It is not clear what the impact of RT is on erectile function after RP, and most men who receive post-RP RT have erectile dysfunction prior to RT [28]. The current study contributes to the existing evidence for post-RP RT by directly examining the influence of RT timing on the occurrence of complication events.

GI events occurred at a higher rate among patients who received SRT than in the ART group (Table 4). Although it is not plausible that prolonged interval between RP and RT would, on its own, cause this increase in events, it is possible that differences in radiation doses used for ART versus SRT may influence risk of GI events. There is evidence that higher SRT doses may result in higher biochemical disease free survival rates [30–32], but may also increase rates of grade 3 and higher GI complications [33]. Since the SEER-Medicare database lacks details regarding radiation doses and technical details, it is not possible for the current study to evaluate whether higher doses were delivered in the SRT group, and this observation warrants additional study.

The study’s external validity is enhanced by the large number of subjects, with a broad range of demographic factors and baseline medical comorbidities. However, there are significant limitations to the study that warrant consideration and attenuate conclusions from this work [34,35]. The SEER-Medicare database lacks details regarding specific intent of the RT courses delivered. Furthermore, there are no data regarding the specifics of the RT fields, and it is possible that some subjects received treatment to sites other than the prostate bed such as metastases. We addressed this issue by limiting the analysis to patients with APFs, who are at higher risk of PC recurrence after RP [4], by excluding patients with a previous primary cancer, and by identifying ART and SRT based upon Medicare claims records that included both procedure codes for RT and an associated diagnostic code for prostate cancer. The study years also spans a time period during which the use of intensity-modulated radiation therapy (IMRT) became more frequent [18], which one might hypothesize would influence the observed rates of events in the current study. However, prior reports have not shown a consistent difference in outcomes between IMRT versus 3-dimensional conformal RT using the SEER-Medicare database [18,19]. The study period also included the publication of the seminal randomized controlled trials of ART versus observation, which one might expect to increase rates of ART delivery. However, two previous reports have shown that the positive results from these trials did not influence the utilization of ART in the SEER database [10,11]. Perhaps most importantly, it should be noted that the procedural codes used to identify events are unlikely to capture mild or moderate side effects of RT that may affect patients’ quality of life related to GU, GI and sexual functional deficits. Therefore, the current comparative effectiveness research study is best viewed as providing evidence that complements the available data from previously published research.

In conclusion, the current study compared outcomes after RP alone, or with the addition of ART or SRT, for a large cohort of men who were eligible for ART after RP based upon the presence of APFs in the surgical specimen. The delivery of RT after RP was associated with increased rates of GI and GU, but not ED, events. Earlier treatment with ART did not increase rates of GU and ED events, when compared to delayed treatment with SRT. RT timing did appear to influence prostate cancer-specific survival. These findings regarding the effect of RT timing on risk of complication events for post-RP prostate cancer patients provide information that may be useful in making decisions regarding delivery of ART and SRT while awaiting the results of ongoing trials of ART versus SRT.

## Supporting Information

S1 Text Additional analytic details of study. Text and explanatory tables pertinent to the analyses are included.
(DOCX)

S1 Table HCPCS and ICD-9-CM procedure and diagnosis codes used for cohort selection and outcome definition. (DOCX)

S2 Table Gastrointestinal events (defined by procedure codes). (DOCX)

S3 Table Erectile dysfunction (defined by procedure codes). (DOCX)

S4 Table Genitourinary incontinence events (defined by procedure codes). (DOCX)

S5 Table Genitourinary non-incontinence events (defined by procedure codes). (DOCX)
