# Supplementary material for: Radiation Therapy after Radical Prostatectomy for Prostate Cancer: Evaluation of Complications and Influence of Radiation Timing on Outcomes in a Large, Population-Based Cohort
Source: PLoS One. 2015 Feb 23;10(2):e0118430. doi: 10.1371/journal.pone.0118430 (PMC4338148; doi:10.1371/journal.pone.0118430)
Supplement: S1 Text — Text and explanatory tables pertinent to the analyses are included. (DOCX) [file pone.0118430.s001.docx]

**Text S1. Additional analytic details of study.**

**Control Variables**

Primary comparisons of complication rates and survival were performed based upon treatment group. Additional characteristics evaluated include individual-level attributes such as race, martial status, year of diagnosis, pathological tumor stage, tumor grade, margin status, age at diagnosis, region of residence, medical co-morbidities, receipt of androgen deprivation therapy, and surgical technique (minimally invasive vs. open), as well as census-tract based factors such as high school education attainment level, median household income, and population density. Additional detail provided in the supplementary material. Radiation therapy dose data or other technical details are not available in the SEER-Medicare database, so the analyses do not reflect adjustment for these factors. Propensity scores were calculated for the receipt of ART, and propensity score weighting was performed to adjust for potential measured confounders.

**Variable descriptions**

Date of Diagnosis (dt_PCdx)

Based on SEER variables: modx1 and yrdx1. No day is provided so date of diagnosis is set to the 1^st^ of the month for all patients.

Date of Death (dod)

Based on SEER variables: ser_dodm and ser_dody. No day is provided so date of death is set to the last day of the month for all patients.

Radiotherapy (RT) (RT, date_RT)

RT was defined based on Medpar, Outpat and NCH files. See Table 2 for a detailed look at HCPCS and ICD-9-CM procedure codes. All modalities of RT were included: IMRT/CRT/Brachy/Proton/NOS.

Prostate-Cancer directed criteria (RT_PC, date_RT_PC)

For patients with only one primary cancer (namely, prostate cancer (PC)) according to SEER, an RT procedure code accompanied by a primary diagnosis code of V580, V1046 or 185 was considered “PC-directed”.

For patients with more than one primary cancer according to SEER, an RT procedure code accompanied by either 1) a primary diagnosis of V580, V1046 AND secondary diagnosis of 185 OR 2) a primary diagnosis of 185, was considered “PC directed”.

Androgen Deprivation Therapy (ADT)

For list of codes see Table 2. ADT use any time during the study period was recorded.

Surgery

For list of codes see Table 2. If both MIRP and ORP codes were present, then the type of surgery was listed as MIRP.

Comorbidity

Comorbidities were coded using ICD-9 diagnosis and procedure codes from Medpar, Outpat and NCH files. The Charlson comorbidity score system [1] was applied using the Deyo ICD-9 code adaptation [2]. Records were extracted from a year prior to diagnosis. Records were extracted if any portion of the record overlapped with the period [date_dx-365, date_dx]. Diagnosis codes were associated with the latter of the admission/from date and the beginning of the 1-year pre-diagnosis period. Procedure codes were associated with the provided procedure date and included if the date fell within the 1-year period.

The metastatic cancer and lymph disease categories were not included in the comorbidity_score_PC calculation since cancer is the disease of interest in this study.

History of Complications (ED, GI, HF, UI, UN) (hx_XX)

Similar to comorbidity, complications were identified using the Medicare claims data. History of complication were extracted from the year prior to RP. ICD-9 procedure codes have a corresponding procedure date. HCPCS cods and ICD-9 diagnosis codes; however, are not associated with specific dates. Thus the admission date (MEDPAR) or from_Date (NCH, OUTPAT) was used for these codes. If this date fell outside of the 1-year window, but the discharge/thru date was after RP-365, then the RP-365 date was used. ICD9 diag date: max(from_date, date_RP-365); ICD9 proc date: proc_datei; HCPCS date: max(proc_datex, RP-365). Basically, HCPCS and ICD-9 diagnosis codes were counted if associated with an admission/record that overlapped in any way with the [date_RP-365,date_RP) window; but ICD-9 procedure codes were only counted if their corresponding procedure date fell within the 1-year window.

Complications (ED, GI, HF, UI, UN) (F_XX)

Uses ICD9 diagnosis, ICD9procedure, and HCPCS codes to identify complications in the Medicare claims data (OUTPAT, NCH, MEDPAR). In order to reduce/eliminate perioperative noise, we started looking for complications starting 30 days after date of RP. Because diagnosis dates are not attached to a particular date, but rather an entire admission (in the case of MEDPAR) or record (for NCH and OUTPAT) we required the admission date (adm_*) in MEDPAR or the from date (from_dt*) in NCH and OUTPAT to be greater than or equal to RP + 30.

Survival time to complication (in days) (survtime_XX)

Time from 30 days post-RT to the first of complication, death and end of study (1JAN2009) in days **plus 1**. We add 1 so that 30 days post-RP counts as the 1^st^ day rather than the 0^th^ day. This shift avoids difficulty arising from taking the log(0) in computing the rate per person years.

Propensity Score

The propensity score was developed via multinomial logistic regression (link = glogit) of ART (vs RP alone or SRT) on the following covariates:

1. race_
2. origrecb
3. marst1
4. education
5. income
6. rural
7. region
8. yrdx
9. pT
10. gleason
11. margins
12. ager1
13. comorbidity_score_PC
14. adt
15. sx_type
16. hx_ed
17. hx_gi
18. hx_hf
19. hx_ui
20. hx_un

Using the method described by Leslie and Thiebaud [3]

**Mapping of Medicare Variables**

| **file** | **pseudonym** | **original** | **combined_date** | **datem** | **dated** | **datey** | **if missing** |
| --- | --- | --- | --- | --- | --- | --- | --- |
| NCH | hcpcs | hcpcs | frexpen_date | frexpenm | frexpend | frexpeny | from_date |
| NCH | diag1 | linediag | frexpen_date | frexpenm | frexpend | frexpeny | from_date |
| NCH | diag2 | pdgns_cd | frexpen_date | frexpenm | frexpend | frexpeny | from_date |
| NCH | diag3 | dgn_cd1 | frexpen_date | frexpenm | frexpend | frexpeny | from_date |
| NCH | diag4 | dgn_cd2 | frexpen_date | frexpenm | frexpend | frexpeny | from_date |
| NCH | diag5 | dgn_cd3 | frexpen_date | frexpenm | frexpend | frexpeny | from_date |
| NCH | diag6 | dgn_cd4 | frexpen_date | frexpenm | frexpend | frexpeny | from_date |
| NCH | diag7 | dgn_cd5 | frexpen_date | frexpenm | frexpend | frexpeny | from_date |
| NCH | diag8 | dgn_cd6 | frexpen_date | frexpenm | frexpend | frexpeny | from_date |
| NCH | diag9 | dgn_cd7 | frexpen_date | frexpenm | frexpend | frexpeny | from_date |
| NCH | diag10 | dgn_cd8 | frexpen_date | frexpenm | frexpend | frexpeny | from_date |
| NCH | proc1 | --- | --- | --- | --- | --- |  |
| NCH | proc2 | --- | --- | --- | --- | --- |  |
| NCH | proc3 | --- | --- | --- | --- | --- |  |
| NCH | proc4 | --- | --- | --- | --- | --- |  |
| NCH | proc5 | --- | --- | --- | --- | --- |  |
| NCH | proc6 | --- | --- | --- | --- | --- |  |
| OUTPAT | hcpcs | hcpcs | from_date | from_dtm | from_dtd | from_dty |  |
| OUTPAT | diag1 | dgn_cd1 | from_date | from_dtm | from_dtd | from_dty |  |
| OUTPAT | diag2 | dgn_cd2 | from_date | from_dtm | from_dtd | from_dty |  |
| OUTPAT | diag3 | dgn_cd3 | from_date | from_dtm | from_dtd | from_dty |  |
| OUTPAT | diag4 | dgn_cd4 | from_date | from_dtm | from_dtd | from_dty |  |
| OUTPAT | diag5 | dgn_cd5 | from_date | from_dtm | from_dtd | from_dty |  |
| OUTPAT | diag6 | dgn_cd6 | from_date | from_dtm | from_dtd | from_dty |  |
| OUTPAT | diag7 | dgn_cd7 | from_date | from_dtm | from_dtd | from_dty |  |
| OUTPAT | diag8 | dgn_cd8 | from_date | from_dtm | from_dtd | from_dty |  |
| OUTPAT | diag9 | dgn_cd9 | from_date | from_dtm | from_dtd | from_dty |  |
| OUTPAT | diag10 | dgn_cd10 | from_date | from_dtm | from_dtd | from_dty |  |
| OUTPAT | proc1 | pr_cd1 | pr_date1 | prdtm1 | prdtd1 | prdty1 | from_date |
| OUTPAT | proc2 | pr_cd2 | pr_date2 | prdtm2 | prdtd2 | prdty2 | from_date |
| OUTPAT | proc3 | pr_cd3 | pr_date3 | prdtm3 | prdtd3 | prdty3 | from_date |
| OUTPAT | proc4 | pr_cd4 | pr_date4 | prdtm4 | prdtd4 | prdty4 | from_date |
| OUTPAT | proc5 | pr_cd5 | pr_date5 | prdtm5 | prdtd5 | prdty5 | from_date |
| OUTPAT | proc6 | pr_cd6 | pr_date6 | prdtm6 | prdtd6 | prdty6 | from_date |
| MEDPAR | hcpcs | --- | --- | --- | --- | --- |  |
| MEDPAR | diag1 | diagcd1 | adm_date | adm_m | adm_d | adm_y |  |
| MEDPAR | diag2 | diagcd2 | adm_date | adm_m | adm_d | adm_y |  |
| MEDPAR | diag3 | diagcd3 | adm_date | adm_m | adm_d | adm_y |  |
| MEDPAR | diag4 | diagcd4 | adm_date | adm_m | adm_d | adm_y |  |
| MEDPAR | diag5 | diagcd5 | adm_date | adm_m | adm_d | adm_y |  |
| MEDPAR | diag6 | diagcd6 | adm_date | adm_m | adm_d | adm_y |  |
| MEDPAR | diag7 | diagcd7 | adm_date | adm_m | adm_d | adm_y |  |
| MEDPAR | diag8 | diagcd8 | adm_date | adm_m | adm_d | adm_y |  |
| MEDPAR | diag9 | diagcd9 | adm_date | adm_m | adm_d | adm_y |  |
| MEDPAR | diag10 | diagcd10 | adm_date | adm_m | adm_d | adm_y |  |
| MEDPAR | proc1 | srgcde1 | srg_date1 | sg1_m | sg1_d | sg1_y | adm_date |
| MEDPAR | proc2 | srgcde2 | srg_date2 | sg2_m | sg2_d | sg2_y | adm_date |
| MEDPAR | proc3 | srgcde3 | srg_date3 | sg3_m | sg3_d | sg3_y | adm_date |
| MEDPAR | proc4 | srgcde4 | srg_date4 | sg4_m | sg4_d | sg4_y | adm_date |
| MEDPAR | proc5 | srgcde5 | srg_date5 | sg5_m | sg5_d | sg5_y | adm_date |
| MEDPAR | proc6 | srgcde6 | srg_date6 | sg6_m | sg6_d | sg6_y | adm_date |

**Diagnosis and Procedure Codes used.**

| **Variable** | **Diagnosis Codes** | **Procedure Codes** | |
| --- | --- | --- | --- |
|  | **ICD-9 Diagnosis** | **ICD-9 Procedure** | **HCPCS** |
| ***Radiation*** |  |  |  |
| IMRT* |  | /*NO CODES*/ | '77301', '77418', '0073T' |
| CRT* |  | '9224', '9226' | '77305', '77310', '77315', '77321', '77371', '77372', '77373', '77402', '77403', '77404', '77406', '77407', '77408', '77409', '77411', '77412', '77413', '77414', '77416', '77422', '77423' |
| Proton* |  | /*NO CODES*/ | '77380', '77381', '77520', '77522', '77523', '77525' |
| Brachytherapy* |  | '9220' | '77326', '77327', '77328', '77776', '77777', '77778', '77781', '77782', '77783', '77784', '77790', '77799', 'Q3001' |
| Original* |  | '922x' | '77399', '77336', '77370', '77301', '77402', '77412', '77417', '77431' |
| ***Androgen Deprivation Therapy*** |  |  |  |
| Orchiectomy |  | '6230', '6241', '6242' | 'G9132', '54520', '54522', '54530', '54535', '54690' |
| Hormones |  | '6240', '6241', '6242' | '54520', '54522', '54530', 54535', '54690', 'J1050', 'J1051', J1950', 'J3315', 'J9202', 'J9217', J9218', 'J9219', 'J9165', 'S0175', C9216', 'C9430', 'G0356', 'J0128', S0165', 'S9560' |
| ***Surgery*** |  |  |  |
| Minimally Invasive RP |  |  | '55866' |
| Open RP |  | '605x' | '55810', '55812', '55815', 55840', '55842', '55845' |

*****Prostate Cancer-directed Criteria applied.

Ex. if _numprims = '01' then do;

if diag1 in ('V580','V1046','185') then do;

rt_orig_pc = **1**;

date_origrt_pc = proc_datex;

end;

end;

if _numprims ne '01' then do;

if (diag1 in ('V580','V1046') and diag2 = '185') then do;

rt_orig_pc = **1**;

date_origrt_pc = proc_datex;

end;

else if (diag1 = '185') then do;

rt_orig_pc = **1**;

date_origrt_pc = proc_datex;

end;

**Supplemental Material References:**

1. Charlson ME, Pompei P, Ales KL, MacKenzie CR. A new method for classifying prognostic comorbidity in longitudinal studies: Development and validation. *J Chron Dis* 1987;40:373-383.

2. Deyo RA, Cherkin DC, Ciol MA. Adapting a clinical comorbidity index for use with icd-9-cm administrative databases. *J Clin Epidemiol* 1992;45:613-619.

3. Leslie S, Thiebaud P. Using propensity scores to adjust for treatment selection bias. *SAS Global Forum Paper* 2007;184.
