# Supplementary material for: Radiation Therapy after Radical Prostatectomy for Prostate Cancer: Evaluation of Complications and Influence of Radiation Timing on Outcomes in a Large, Population-Based Cohort
Source: PLoS One. 2015 Feb 23;10(2):e0118430. doi: 10.1371/journal.pone.0118430 (PMC4338148; doi:10.1371/journal.pone.0118430)
Supplement: S1 Table — (DOCX) [file pone.0118430.s002.docx]

**Table S1.** **HCPCS and ICD-9-CM procedure and diagnosis codes used for cohort selection and outcome definition.**

| **Variable** | **Diagnosis Codes** | **Procedure Codes** | |
| --- | --- | --- | --- |
|  | **ICD-9 Diagnosis** | **ICD-9 Procedure** | **HCPCS** |
| ***Radiation*** |  | 92.2x | 77301, 77305, 77310, 77315, 77321,  77326, 77327, 77328, 77336, 77370, 77371, 77372, 77373, 77380, 77381, 77399, 77402, 77403, 77404, 77406, 77407, 77408, 77409, 77411, 77412, 77413, 77414, 77416, 77417, 77418, 77422, 77423, 77431, 77520, 77522, 77523, 77525, 77776, 77777, 77778, 77781, 77782, 77783, 77784, 77790,  77799, 0073T, Q3001 |
| ***Androgen Deprivation Therapy*** |  | 62.30, 62.40, 62.41, 62.42 | 54520, 54522, 54530, 54535, 54690, C9216, C9430, G0356, G9132, J0128, J1050, J1051, J1950, J3315, J9165, J9202, J9217, J9218, J9219, S0165, S0175, S9560 |
| ***Surgery*** |  |  |  |
| Minimally Invasive RP |  |  | 55866 |
| Open RP |  | 60.5x | 55810, 55812, 55815, 55840, 55842, 55845 |
| **Complications** |  |  |  |
| Gastrointestinal | 555-558, 558.1, 560.81, 560.89, 560.9, 564.5, 565, 566, 569.2-569.4, 569.41, 569.8, 569.81, 578, 578.9, 787.91 | 45.23, 45.25, 46.03, 46.1x, 48.23, 48.24, 48.31-48.33, 48.4-48.7, 48.62, 48.73, 48.9, 48.93, 49.1, 49.21, 49.23, 49.6, 49.7, 49.73, 93.95, 96.22, 96.23 | 44140-44160, 44204-44212, 45000, 45005, 45020, 45110-45123, 45300-45345, 45355-45387, 45500, 45562, 45563, 45800-45805, 45820-45825, 45905, 45910, 46600-46615, 46700, 99183, G0167 |
| Genitourinary Incontinence | 596.1, 596.2, 599.1, 599.82, 788.3x | 59.3-59.6, 57.83, 57.84, 58.43, 58.93, 59.71, 59.72, 59.79, 89.21-89.25 | 44660, 44661, 51715, 51725, 51726, 51736, 51741, 51772, 51784, 51785, 51792, 51795, 51797, 51798, 51840, 51841, 53440, 53442, 53443, 53445, 53447, 53520 |
| Genitourinary non-incontinence | 595.82, 596.0, 596.7, 598.x, 599.6, 788.2x | 57.85, 57.91-57.93, 58.0, 58.1, 58.3x, 58.44, 58.46, 58.47, 58.5, 58.6, 58.99, 60.2x, 60.95 | 52275, 52276, 52281-52283, 52510, 52601, 52612, 52614, 52620, 52630, 53010, 53400, 53405, 53410, 53415, 53420, 53425, 53600, 53601, 53605, 53620, 53621, 53850, 53852 |
| **Variable** | **Diagnosis Codes** | **Procedure Codes** | |
|  | **ICD-9 Diagnosis** | **ICD-9 Procedure** | **HCPCS** |
| Erectile dysfunction | 607.84 | 64.94-64.97 | 54231, 54235, 54400-54402, 54405-54411, 54415-54417, C1007, C1813, C2622, C3500, C8514, C8516, C8534, J0270, J0275, J2440, J2760, L7900 |
