# Supplementary material for: Radiation Therapy after Radical Prostatectomy for Prostate Cancer: Evaluation of Complications and Influence of Radiation Timing on Outcomes in a Large, Population-Based Cohort
Source: PLoS One. 2015 Feb 23;10(2):e0118430. doi: 10.1371/journal.pone.0118430 (PMC4338148; doi:10.1371/journal.pone.0118430)
Supplement: S2 Table — (DOCX) [file pone.0118430.s003.docx]

**Table S2. Gastrointestinal events (defined by procedure codes)**

| **Predictor** | **HR** | **95% CI** | **p** | **Global p** |
| --- | --- | --- | --- | --- |
| **Radiotherapy Use** |  |  |  | <0.001 |
| ART (<9mo) vs. RP alone | 1.17 | (1.06, 1.29) | 0.003 |  |
| SRT (12mo+) vs. RP alone | 1.46 | (1.25, 1.69) | <0.001 |  |
| **Pathological T-Stage** |  |  |  | 0.916 |
| T3a vs. T2 | 1.01 | (0.90, 1.12) | 0.905 |  |
| T3b vs. T2 | 1.03 | (0.88, 1.20) | 0.718 |  |
| **Gleason Score** |  |  |  | 0.102 |
| 8+ vs. ≤7 | 0.94 | (0.86, 1.01) | 0.102 |  |
| **Margins Status** |  |  |  | 0.897 |
| Involved vs. Uninvolved | 1.01 | (0.91, 1.12) | 0.897 |  |
| **Age at Diagnosis** |  |  |  | 0.840 |
| 70-74 vs. 66-69 | 0.97 | (0.90, 1.05) | 0.419 |  |
| 75-79 vs. 66-69 | 1.00 | (0.87, 1.15) | 0.976 |  |
| 80+ vs. 66-69 | 0.91 | (0.57, 1.45) | 0.676 |  |
| **Radical Prostatectomy Type** |  |  |  | 0.106 |
| MIRP vs. Open | 1.12 | (0.98, 1.29) | 0.106 |  |
| **Androgen Deprivation Therapy** |  |  |  | 0.990 |
| Yes vs. No | 1.00 | (0.92, 1.09) | 0.990 |  |
| **Race** |  |  |  | 0.999 |
| Black vs. White | 1.00 | (0.85, 1.18) | 0.995 |  |
| Other/Unspecified vs. White | 1.00 | (0.84, 1.19) | 0.958 |  |
| **Hispanic Ethnicity** |  |  |  | 0.003 |
| Hispanic vs. Non-Hispanic | 0.77 | (0.65, 0.91) | 0.003 |  |
| **Median Household Income** |  |  |  | 0.009 |
| 35K-44K vs. <35K | 1.09 | (0.97, 1.23) | 0.167 |  |
| 45K-59K vs. <35K | 1.20 | (1.05, 1.36) | 0.006 |  |
| 60K+ vs. <35K | 1.27 | (1.10, 1.46) | 0.001 |  |
| **Treatment Region** |  |  |  | 0.936 |
| Midwest vs. West | 1.00 | (0.91, 1.10) | 0.976 |  |
| Northeast vs. West | 1.04 | (0.91, 1.18) | 0.576 |  |
| South vs. West | 0.98 | (0.87, 1.12) | 0.802 |  |
| **Year of Diagnosis** |  |  |  | 0.489 |
| 2000-2004 vs. 1995-1999 | 1.05 | (0.96, 1.15) | 0.264 |  |
| 2005-2007 vs. 1995-1999 | 1.01 | (0.89, 1.15) | 0.859 |  |
| **Marital Status** |  |  |  | 0.164 |
| Married vs. Not Married | 1.06 | (0.95, 1.18) | 0.278 |  |
| Unknown vs. Not Married | 1.25 | (0.99, 1.59) | 0.063 |  |
| **HS Education Attainment** |  |  |  | 0.040 |
| 75-84.99% vs. <75% | 1.03 | (0.90, 1.17) | 0.684 |  |
| 85-89.99% vs. <75% | 0.92 | (0.79, 1.06) | 0.227 |  |
| 90%+ vs. <75% | 1.07 | (0.92, 1.24) | 0.401 |  |
| **Predictor** | **HR** | **95% CI** | **p** | **Global p** |
| **Population Density** |  |  |  | 0.114 |
| Rural vs. Urban | 0.79 | (0.58, 1.06) | 0.114 |  |
| **Comorbidity Score** |  |  |  | 0.439 |
| 1 vs. 0 | 1.05 | (0.97, 1.14) | 0.235 |  |
| 2+ vs. 0 | 0.99 | (0.88, 1.11) | 0.834 |  |
| **History of ED** |  |  |  | 0.147 |
| Yes vs. No | 0.92 | (0.81, 1.03) | 0.147 |  |
| **History of GI** |  |  |  | <0.001 |
| Yes vs. No | 1.17 | (1.07, 1.27) | <0.001 |  |
| **History of UI** |  |  |  | <0.001 |
| Yes vs. No | 1.31 | (1.18, 1.47) | <0.001 |  |
| **History of UN** |  |  |  | 0.045 |
| Yes vs. No | 0.90 | (0.81, 1.00) | 0.045 |  |
