# Supplementary material for: Radiation Therapy after Radical Prostatectomy for Prostate Cancer: Evaluation of Complications and Influence of Radiation Timing on Outcomes in a Large, Population-Based Cohort
Source: PLoS One. 2015 Feb 23;10(2):e0118430. doi: 10.1371/journal.pone.0118430 (PMC4338148; doi:10.1371/journal.pone.0118430)
Supplement: S3 Table — (DOCX) [file pone.0118430.s004.docx]

**Table S3. Erectile dysfunction (defined by procedure codes).**

| **Predictor** | **HR** | **95% CI** | **p** | **Global p** |
| --- | --- | --- | --- | --- |
| **Radiotherapy Use** |  |  |  | 0.267 |
| ART (<9mo) vs. RP alone | 0.99 | (0.78, 1.24) | 0.896 |  |
| SRT (12mo+) vs. RP alone | 0.54 | (0.26, 1.14) | 0.104 |  |
| **Pathological T-Stage** |  |  |  | 0.507 |
| T3a vs. T2 | 1.03 | (0.82, 1.31) | 0.783 |  |
| T3b vs. T2 | 1.18 | (0.84, 1.65) | 0.332 |  |
| **Gleason Score** |  |  |  | 0.312 |
| 8+ vs. ≤7 | 0.91 | (0.76, 1.09) | 0.312 |  |
| **Margins Status** |  |  |  | 0.676 |
| Involved vs. Uninvolved | 1.05 | (0.84, 1.32) | 0.676 |  |
| **Age at Diagnosis** |  |  |  | <0.001 |
| 70-74 vs. 66-69 | 0.79 | (0.67, 0.93) | 0.004 |  |
| 75-79 vs. 66-69 | 0.59 | (0.42, 0.83) | 0.002 |  |
| 80+ vs. 66-69 | 0.30 | (0.06, 1.45) | 0.134 |  |
| **Radical Prostatectomy Type** |  |  |  | 0.824 |
| MIRP vs. Open | 1.03 | (0.79, 1.36) | 0.824 |  |
| **Androgen Deprivation Therapy** |  |  |  | 0.437 |
| Yes vs. No | 0.93 | (0.77, 1.12) | 0.437 |  |
| **Race** |  |  |  | <0.001 |
| Black vs. White | 1.54 | (1.14, 2.08) | 0.005 |  |
| Other/Unspecified vs. White | 0.47 | (0.26, 0.83) | 0.010 |  |
| **Hispanic Ethnicity** |  |  |  | 0.024 |
| Hispanic vs. Non-Hispanic | 1.43 | (1.05, 1.94) | 0.024 |  |
| **Median Household Income** |  |  |  | 0.054 |
| 35K-44K vs. <35K | 1.00 | (0.77, 1.28) | 0.975 |  |
| 45K-59K vs. <35K | 0.73 | (0.55, 0.97) | 0.030 |  |
| 60K+ vs. <35K | 0.91 | (0.67, 1.25) | 0.572 |  |
| **Treatment Region** |  |  |  | <0.001 |
| Midwest vs. West | 0.68 | (0.53, 0.86) | 0.001 |  |
| Northeast vs. West | 0.49 | (0.34, 0.71) | <0.001 |  |
| South vs. West | 1.07 | (0.83, 1.38) | 0.588 |  |
| **Year of Diagnosis** |  |  |  | 0.049 |
| 2000-2004 vs. 1995-1999 | 0.84 | (0.70, 1.03) | 0.087 |  |
| 2005-2007 vs. 1995-1999 | 0.73 | (0.57, 0.95) | 0.017 |  |
| **Marital Status** |  |  |  | 0.988 |
| Married vs. Not Married | 1.02 | (0.81, 1.27) | 0.886 |  |
| Unknown vs. Not Married | 1.03 | (0.60, 1.78) | 0.912 |  |
| **HS Education Attainment** |  |  |  | 0.279 |
| 75-84.99% vs. <75% | 1.29 | (0.98, 1.69) | 0.072 |  |
| 85-89.99% vs. <75% | 1.23 | (0.89, 1.69) | 0.205 |  |
| 90%+ vs. <75% | 1.34 | (0.96, 1.86) | 0.086 |  |
| **Predictor** | **HR** | **95% CI** | **p** | **Global p** |
| **Population Density** |  |  |  | 0.509 |
| Rural vs. Urban | 1.22 | (0.67, 2.21) | 0.509 |  |
| **Comorbidity Score** |  |  |  | 0.011 |
| 1 vs. 0 | 0.85 | (0.71, 1.02) | 0.083 |  |
| 2+ vs. 0 | 0.68 | (0.52, 0.89) | 0.006 |  |
| **History of ED** |  |  |  | <0.001 |
| Yes vs. No | 2.11 | (1.72, 2.58) | <0.001 |  |
| **History of GI** |  |  |  | 0.013 |
| Yes vs. No | 1.26 | (1.05, 1.51) | 0.013 |  |
| **History of UI** |  |  |  | 0.002 |
| Yes vs. No | 1.44 | (1.15, 1.80) | 0.002 |  |
| **History of UN** |  |  |  | 0.038 |
| Yes vs. No | 1.24 | (1.01, 1.53) | 0.038 |  |
