# Supplementary material for: Radiation Therapy after Radical Prostatectomy for Prostate Cancer: Evaluation of Complications and Influence of Radiation Timing on Outcomes in a Large, Population-Based Cohort
Source: PLoS One. 2015 Feb 23;10(2):e0118430. doi: 10.1371/journal.pone.0118430 (PMC4338148; doi:10.1371/journal.pone.0118430)
Supplement: S4 Table — (DOCX) [file pone.0118430.s005.docx]

**Table S4. Genitourinary incontinence events (defined by procedure codes)**

| **Predictor** | **HR** | **95% CI** | **p** | **Global p** |
| --- | --- | --- | --- | --- |
| **Radiotherapy Use** |  |  |  | <0.001 |
| ART (<9mo) vs. RP alone | 1.22 | (1.05, 1.41) | 0.008 |  |
| SRT (12mo+) vs. RP alone | 1.40 | (1.12, 1.76) | 0.004 |  |
| **Pathological T-Stage** |  |  |  | 0.796 |
| T3a vs. T2 | 0.96 | (0.83, 1.12) | 0.615 |  |
| T3b vs. T2 | 0.93 | (0.76, 1.15) | 0.499 |  |
| **Gleason Score** |  |  |  | 0.404 |
| 8+ vs. ≤7 | 0.96 | (0.86, 1.06) | 0.404 |  |
| **Margins Status** |  |  |  | 0.243 |
| Involved vs. Uninvolved | 0.92 | (0.80, 1.06) | 0.243 |  |
| **Age at Diagnosis** |  |  |  | 0.220 |
| 70-74 vs. 66-69 | 1.10 | (0.99, 1.21) | 0.075 |  |
| 75-79 vs. 66-69 | 1.05 | (0.88, 1.26) | 0.600 |  |
| 80+ vs. 66-69 | 0.75 | (0.42, 1.35) | 0.336 |  |
| **Radical Prostatectomy Type** |  |  |  | 0.528 |
| MIRP vs. Open | 0.95 | (0.81, 1.12) | 0.528 |  |
| **Androgen Deprivation Therapy** |  |  |  | <0.001 |
| Yes vs. No | 1.24 | (1.11, 1.38) | <0.001 |  |
| **Race** |  |  |  | 0.863 |
| Black vs. White | 0.98 | (0.80, 1.20) | 0.846 |  |
| Other/Unspecified vs. White | 0.94 | (0.74, 1.19) | 0.607 |  |
| **Hispanic Ethnicity** |  |  |  | 0.014 |
| Hispanic vs. Non-Hispanic | 1.26 | (1.05, 1.52) | 0.014 |  |
| **Median Household Income** |  |  |  | 0.048 |
| 35K-44K vs. <35K | 0.95 | (0.81, 1.11) | 0.497 |  |
| 45K-59K vs. <35K | 1.05 | (0.89, 1.25) | 0.546 |  |
| 60K+ vs. <35K | 1.20 | (0.99, 1.46) | 0.063 |  |
| **Treatment Region** |  |  |  | 0.174 |
| Midwest vs. West | 0.92 | (0.80, 1.06) | 0.243 |  |
| Northeast vs. West | 1.05 | (0.89, 1.25) | 0.557 |  |
| South vs. West | 1.13 | (0.96, 1.33) | 0.134 |  |
| **Year of Diagnosis** |  |  |  | <0.001 |
| 2000-2004 vs. 1995-1999 | 1.37 | (1.20, 1.56) | <0.001 |  |
| 2005-2007 vs. 1995-1999 | 1.97 | (1.68, 2.32) | <0.001 |  |
| **Marital Status** |  |  |  | 0.584 |
| Married vs. Not Married | 0.93 | (0.82, 1.07) | 0.309 |  |
| Unknown vs. Not Married | 0.97 | (0.71, 1.33) | 0.857 |  |
| **HS Education Attainment** |  |  |  | 0.069 |
| 75-84.99% vs. <75% | 0.88 | (0.74, 1.03) | 0.114 |  |
| 85-89.99% vs. <75% | 0.86 | (0.72, 1.04) | 0.121 |  |
| 90%+ vs. <75% | 0.77 | (0.63, 0.94) | 0.009 |  |
| **Predictor** | **HR** | **95% CI** | **p** | **Global p** |
| **Population Density** |  |  |  | 0.379 |
| Rural vs. Urban | 0.83 | (0.55, 1.26) | 0.379 |  |
| **Comorbidity Score** |  |  |  | <0.001 |
| 1 vs. 0 | 1.18 | (1.06, 1.32) | 0.003 |  |
| 2+ vs. 0 | 1.38 | (1.19, 1.58) | <0.001 |  |
| **History of ED** |  |  |  | 0.017 |
| Yes vs. No | 1.19 | (1.03, 1.37) | 0.017 |  |
| **History of GI** |  |  |  | 0.002 |
| Yes vs. No | 1.19 | (1.06, 1.33) | 0.002 |  |
| **History of UI** |  |  |  | <0.001 |
| Yes vs. No | 2.27 | (2.00, 2.57) | <0.001 |  |
| **History of UN** |  |  |  | <0.001 |
| Yes vs. No | 1.28 | (1.13, 1.44) | <0.001 |  |
