# Supplementary material for: Radiation Therapy after Radical Prostatectomy for Prostate Cancer: Evaluation of Complications and Influence of Radiation Timing on Outcomes in a Large, Population-Based Cohort
Source: PLoS One. 2015 Feb 23;10(2):e0118430. doi: 10.1371/journal.pone.0118430 (PMC4338148; doi:10.1371/journal.pone.0118430)
Supplement: S5 Table — (DOCX) [file pone.0118430.s006.docx]

**Table S5. Genitourinary non-incontinence events (defined by procedure codes).**

| **Predictor** | **HR** | **95% CI** | **p** | **Global p** |
| --- | --- | --- | --- | --- |
| **Radiotherapy Use** |  |  |  | <0.001 |
| ART (<9mo) vs. RP alone | 1.71 | (1.45, 2.01) | <0.001 |  |
| SRT (12mo+) vs. RP alone | 1.42 | (1.02, 1.97) | 0.039 |  |
| **Pathological T-Stage** |  |  |  | 0.956 |
| T3a vs. T2 | 1.02 | (0.86, 1.23) | 0.794 |  |
| T3b vs. T2 | 1.01 | (0.79, 1.30) | 0.927 |  |
| **Gleason Score** |  |  |  | 0.865 |
| 8+ vs. ≤7 | 0.99 | (0.87, 1.13) | 0.865 |  |
| **Margins Status** |  |  |  | 0.982 |
| Involved vs. Uninvolved | 1.00 | (0.85, 1.19) | 0.982 |  |
| **Age at Diagnosis** |  |  |  | 0.451 |
| 70-74 vs. 66-69 | 1.08 | (0.96, 1.23) | 0.199 |  |
| 75-79 vs. 66-69 | 1.15 | (0.93, 1.41) | 0.210 |  |
| 80+ vs. 66-69 | 1.15 | (0.60, 2.21) | 0.668 |  |
| **Radical Prostatectomy Type** |  |  |  | <0.001 |
| MIRP vs. Open | 0.42 | (0.31, 0.57) | <0.001 |  |
| **Androgen Deprivation Therapy** |  |  |  | <0.001 |
| Yes vs. No | 1.28 | (1.12, 1.46) | <0.001 |  |
| **Race** |  |  |  | 0.067 |
| Black vs. White | 0.72 | (0.54, 0.95) | 0.020 |  |
| Other/Unspecified vs. White | 0.96 | (0.71, 1.28) | 0.764 |  |
| **Hispanic Ethnicity** |  |  |  | 0.650 |
| Hispanic vs. Non-Hispanic | 1.06 | (0.83, 1.34) | 0.650 |  |
| **Median Household Income** |  |  |  | 0.215 |
| 35K-44K vs. <35K | 1.14 | (0.94, 1.38) | 0.195 |  |
| 45K-59K vs. <35K | 1.24 | (1.01, 1.51) | 0.041 |  |
| 60K+ vs. <35K | 1.24 | (0.98, 1.56) | 0.069 |  |
| **Treatment Region** |  |  |  | 0.310 |
| Midwest vs. West | 0.86 | (0.73, 1.02) | 0.075 |  |
| Northeast vs. West | 0.90 | (0.72, 1.13) | 0.376 |  |
| South vs. West | 0.94 | (0.76, 1.16) | 0.571 |  |
| **Year of Diagnosis** |  |  |  | 0.001 |
| 2000-2004 vs. 1995-1999 | 0.84 | (0.73, 0.97) | 0.014 |  |
| 2005-2007 vs. 1995-1999 | 0.65 | (0.53, 0.79) | <0.001 |  |
| **Marital Status** |  |  |  | 0.009 |
| Married vs. Not Married | 0.78 | (0.67, 0.92) | 0.002 |  |
| Unknown vs. Not Married | 0.78 | (0.50, 1.19) | 0.248 |  |
| **HS Education Attainment** |  |  |  | 0.035 |
| 75-84.99% vs. <75% | 0.82 | (0.67, 1.00) | 0.050 |  |
| 85-89.99% vs. <75% | 0.81 | (0.65, 1.02) | 0.069 |  |
| **Predictor** | **HR** | **95% CI** | **p** | **Global p** |
| 90%+ vs. <75% | 0.71 | (0.56, 0.89) | 0.004 |  |
| **Population Density** |  |  |  | 0.013 |
| Rural vs. Urban | 0.41 | (0.21, 0.83) | 0.013 |  |
| **Comorbidity Score** |  |  |  | 0.480 |
| 1 vs. 0 | 1.03 | (0.90, 1.18) | 0.681 |  |
| 2+ vs. 0 | 1.12 | (0.93, 1.34) | 0.228 |  |
| **History of ED** |  |  |  | 0.176 |
| Yes vs. No | 0.87 | (0.70, 1.07) | 0.176 |  |
| **History of GI** |  |  |  | 0.024 |
| Yes vs. No | 1.17 | (1.02, 1.34) | 0.024 |  |
| **History of UI** |  |  |  | 0.063 |
| Yes vs. No | 1.19 | (0.99, 1.42) | 0.063 |  |
| **History of UN** |  |  |  | <0.001 |
| Yes vs. No | 1.37 | (1.17, 1.59) | <0.001 |  |
